# Predicting Drug–Target Interactions Based on the Ensemble Models of Multiple Feature Pairs

**DOI:** 10.3390/ijms22126598

**Published:** 2021-06-20

**Authors:** Cheng Wang, Jun Zhang, Peng Chen, Bing Wang

**Affiliations:** 1 Department of Computer Science & Technology, Tongji University, Shanghai 201804, China; wangcheng0788@tongji.edu.cn; 2 Institutes of Physical Science and Information Technology & School of Internet, Anhui University, Hefei 230601, China; 00568@ahu.edu.cn; 3 School of Electrical & Information Engineering, Anhui University of Technology, Ma’anshan 243032, China; 4 Key Laboratory of Power Electronics and Motion Control Anhui Education Department, Ma’anshan 243032, China

**Keywords:** drug–target interactions, ensemble model of Multiple Feature Pairs (Ensemble-MFP), model weight sum, support vector machines

## Abstract

Backgroud: The prediction of drug–target interactions (DTIs) is of great significance in drug development. It is time-consuming and expensive in traditional experimental methods. Machine learning can reduce the cost of prediction and is limited by the characteristics of imbalanced datasets and problems of essential feature selection. Methods: The prediction method based on the Ensemble model of Multiple Feature Pairs (Ensemble-MFP) is introduced. Firstly, three negative sets are generated according to the Euclidean distance of three feature pairs. Then, the negative samples of the validation set/test set are randomly selected from the union set of the three negative sets in the validation set/test set. At the same time, the ensemble model with weight is optimized and applied to the test set. Results: The area under the receiver operating characteristic curve (area under ROC, AUC) in three out of four sub-datasets in gold standard datasets was more than 94.0% in the prediction of new drugs. The effectiveness of the proposed method is also shown with the comparison of state-of-the-art methods and demonstration of predicted drug–target pairs. Conclusion: The Ensemble-MFP can weigh the existing feature pairs and has a good prediction effect for general prediction on new drugs.

## 1. Introduction

The prediction of drug–target Interaction (DTI) based on machine learning is very important in pharmacology and drug design [1,2,3]. It can also be considered as one direction in chemogenomics, which is a new interdisciplinary subject of biology, chemistry and informatics [4,5]. Traditional DTI methods are time-consuming, costly, and make it difficult to obtain three-dimensional structures of compounds and proteins [6,7,8]. The technology of machine learning accelerates the development of drug–target interactions, especially in reducing the blindness of experiments [9,10,11,12,13].

The characteristics of imbalance datasets in drug–target interaction predictions restrict the development of machine learning [1,14,15,16,17,18]. In the datasets of DTIs, the drug–target pairs with identified interactions which are labeled positive are sparse. At the same time, there are no validated negative samples, that is, non-interaction, in most databases [19]. In other words, the datasets of DTIs cannot provide enough reliable positive and negative samples for machine learning to obtain stable models [20]. To solve this problem, extraction methods of negative samples were studied. The random sampling method is used for negative extraction in various papers, which randomly selects negative samples from unlabeled sets [1,21]. Other negative sampling methods were also discussed. Liu et al. assumed that the negative samples can be extracted by their dissimilar characteristics from positive ones [22]. Hu et al. introduced the method based on Euclidean distance for negative sampling, and obtained better predictions [7]. Moderlet et al. introduced a bootstrap aggregating technique for negative sampling in Positive-Unlabeled (PU) problems [23].

The effective feature pairs selection in DTIs is another problem that restricts machine learning [1,24]. There are many types of features that can describe the characteristics of drugs or target proteins. The feature pair of DTI can be defined as the combination of one or more drug descriptors and one or more target descriptors. The dimensions of drug descriptors and target descriptors can be different or the same according to the feature extraction methods. Researchers have explored many types of feature pairs to predict drug–target interactions. Wei et al. predicted the interactions combined with 881-dimensional drug-descriptors, and target-descriptors of 567-dimensionals and 1449-dimensionals [25]. Bahi et al. combined 193-dimensional drug-descriptors based on the RCPI package and 1290-dimensional target-descriptors from PROFEAT to predict the interactions [11]. Feng et al. proposed the Deep Belief Network (DBN) for DTI based on 6144-dimensional Extended-Connectivity Fingerprints (ECFP) of drugs, and the 8420-dimensional Protein Sequence Composition (PSC) [26].

The prediction based on the Ensemble models of Multiple Feature Pairs (Ensemble-MFP) for new drugs prediction is studied in this work. Negative sampling based on the Euclidean distance, which is used to obtain the most dissimilar samples compared with positive sampling, is highly dependent on the calculated feature pairs, and in particular, the prediction of negative sampling based on different feature pairs is more prone to bias. At the same time, considering that the basic feature pairs of DTI are not clear and it is difficult to discover new feature pairs, an ensemble of the models based on existing feature pairs to have better predictions is necessary. The construction of a validation set and test set is designed to ensure the generalization ability of the algorithm and avoid the problem of overfitting. The final model is the weighted sum of sub-models corresponding to three feature pairs in this work, and the weights are optimized. Finally, the results on the test sets show that the algorithm is effective. Through the prediction of independent datasets by the proposed model, some drug–target pairs with interactions were predicted, which shows that the ensemble model has a good predictive effect on new drugs (see Appendix C). At the same time, we also provide several groups of drug–target pairs that may have interactions for further research in wet-lab. It should be noted that although the research regards the drug–target interaction as a binary classification problem, the actual situation is more complex with the strength of interactions, inhibitor or agonist, and so forth. Therefore, our model has limitations in broader predictions.

## 2. Materials and Methods

### 2.1. Benchmark Datasets

The benchmark dataset used in this work is the Gold Standard Dataset, which was first introduced by Yamanishi et al. It was collected and constructed in 2008, from KEGG BRITE, DrugBank, BRENDA, and SuperTarget [19,27,28,29,30]. According to the different characteristics of the target protein, it was divided into four sub-datasets: the enzyme, GPCR, ion channel, and nuclear receptor. It is publicly available on http://web.kuicr.kyoto-u.ac.jp/supp/yoshi/drugtarget/, accessed on 1 July 2008. Table 1 shows their statistical information in detail. It can be seen from the Table that the number of positive samples is far less than that of unlabeled samples, that is, the data are seriously imbalanced. It is very important that the prediction research needs reliable negative sample information.

### 2.2. Evaluation Criteria

The Area Under the Curve for the receiver operating characteristic (Area Under ROC, AUC), is the performance criteria used in this work. The metrics, such as Accuracy, Precision, Recall, and so forth are sufficient in general classification problems, but hold no significance in imbalanced datasets [1]. Some of the parameters used for evaluation are calculated as follows:

(1) $$Accuracy=\frac{TP+TN}{TP+TN+FP+FN}$$

(2) $$Precision=\frac{TP}{TP+FP}$$

(3) $$Recall=\frac{TP}{TP+FN}$$

(4) $$F1\_score=\frac{2\times Precision\times Recall}{Precision+Recall},$$

where *TP*, *TN*, *FP*, and *FN* are true positive, true negative, false positive and false negative, respectively. In these parameters, “positive” and “negative” represent drug–target pairs labeled as interaction or non-interaction in the benchmark dataset. At the same time, “true” and “false” mean that the prediction of the drug–target pair is right or wrong. ROC curves are drawn according to the True Positive Rate (TPR) and False Positive Rate (FPR) of different thresholds in the classification, and are recommended for comprehensive evaluation, especially in imbalance classification. AUC is the area under the ROC curve, and can be easily compared. It ranges from 0 to 1, and the larger the value, the better the model. Application of AUC can be found in most papers related with classification [1,17,31,32,33].

### 2.3. Negative Sampling and Data Construction

Negative samples are mainly generated based on the Euclidean distance in this work. Different from the random sampling method, the Euclidean distance-based sampling method holds that the farther the sample is from the positive center, the more reliable the negative sample is [7]. Its formula is as follows:

(5) $$Dis=\sqrt{\sum {\left(pos_{d,t}-unlabel_{d,t}\right)}^{2}},$$

where $pos_{d,t}$ denotes the positive samples’ center of the mean calculation. $unlabel_{d,t}$ denotes the unlabeled samples. All unlabeled samples will be sorted according to their distance from the center of positive samples (Dis). The larger the Dis is, the more reliable the negative samples are. In order to avoid the negative sample difference caused by different feature units, all features used are firstly normalized. At the same time, Principal Components Analysis (PCA) is performed to avoid the interference of correlation in the calculation of the Euclidean distance. Although the sampling method is effective, it has a high dependence on the selected feature pairs and is difficult to be generalized, especially for the negative samples generated by different feature pairs, or is selected randomly. In order to improve the generalization ability of the model and obtain better prediction results, this work designs an ensemble model method of multiple feature pairs.

**Figure 1:**
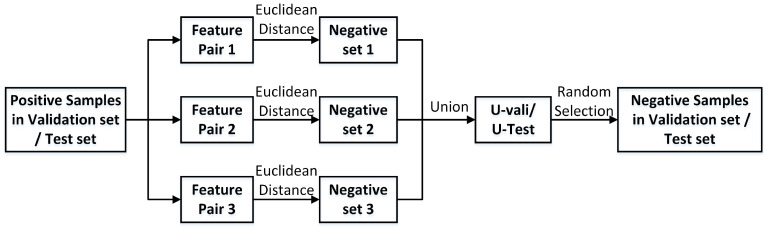
The negative sampling process of the validation set and test set.

Data construction is based on 5-fold cross-validation. In order to make the model reliable for new drugs, the drugs in the dataset are divided with the ratio of 0.6, 0.2, and 0.2, respectively. In other words, DT pairs are divided into the training set, validation set, and test set according to different drugs. This work uses three feature pairs to get three corresponding models, and generates more general negative samples in the validation set and test set. The negative sampling process of the validation set and test set is shown in Figure 1.

**Figure 2:**
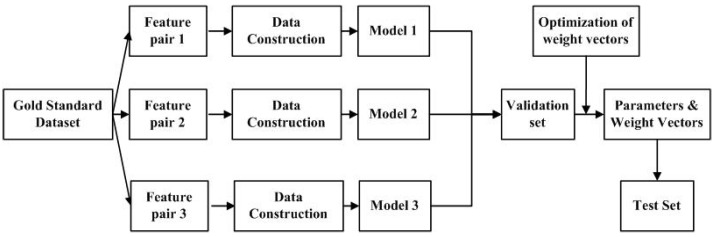
The flowchart of the Ensemble-MFP algorithm.

Firstly, in the validation set/test set, based on Euclidean distance calculation, three feature pairs are used for negative sampling. Then, the negative samples were combined into *U-vali*/*U-test*. Random selection from *U-vali*/*U-test* can get more general negative samples for the validation set/test set. For the training set, the three feature pairs are trained respectively by the method of negative sampling based on Euclidean distance, and three models are obtained. According to these three models, the validation set is weighted and optimized to get a better weight vector, which is applied to the test set. The calculation formula of ensemble models is as follows,

(6) $$dec=\sum w_{i}\times dec_{i},$$

where $w_{i}$ represents the weight of feature pair *i*. $dec_{i}$ represents the decision vectors predicted by $model_{i}$. dec and $model_{i}$ denote the optimized decision vector in the validation set/test set and the model trained according with feature pair *i*. The flowchart of the proposed algorithm is shown in Figure 2.

### 2.4. Feature Pairs and Algorithm

Feature pairs used in Ensemble-MFP are extracted from the PaDEL-Descriptor and PROFEAT. The PaDEL-Descriptor is a free software for generating drug-descriptors, and is available on https://www.winsite.com/, accessed on 12 October 2010 [34]. PROFEAT is a webservice for calculation protein features, and can be used on http://bidd.group/, accessed on 12 April 2011 [35,36]. Table 2 lists the feature pairs used in the proposed method, which have better predictability in all sub-datasets of the gold standard dataset. In the Table, Estate-FP, MACCS-FP and Sub-FP Count are shorts for Electrotopological State Fingerprints, MACCS Fingerprints, and the Substructure Fingerprints Count, respectively. AAC, APAAC and QSO are short for Amino Acid Composition, Amphiphilic Pseudo-Amino Acid Composition, and Quasi-Sequence-Order descriptors, respectively.

Support vector machines (SVM) and its toolbox Libsvm (version 3.23) are adopted in this work. The Radial Basis Function (RBF) kernel, which can easily process the nonlinear classification problems, is used, and the kernel function only needs to adjust two parameters, *c* and γ. The parameters are adjusted in the form of an exponent, with the bottom of 2 [37]. Finally, the optimized parameters have good performance in four sub-datasets, that is, $c=2^{-4}$ and $\gamma =2^{-7}$.

## 3. Results

### 3.1. Performance on DTIs

**Figure 3:**
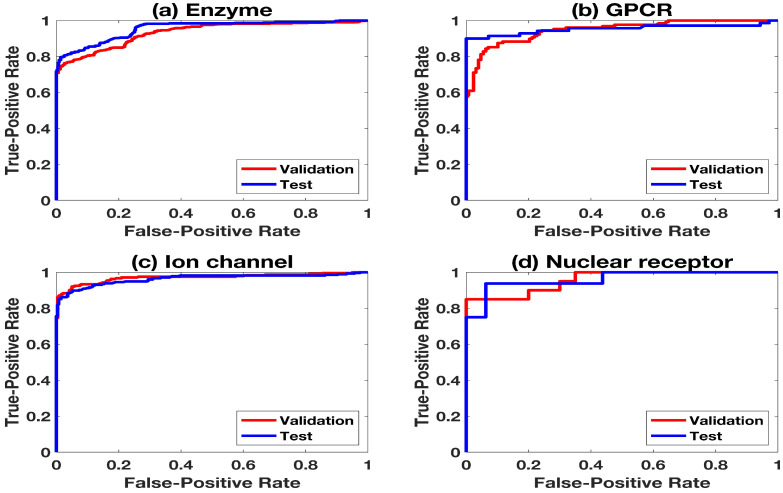
ROC-curves predicted on the validation set and the test set of each sub-dataset. The experiments are based on different drugs of the training set, validation set, and test set.

**Figure A1:**
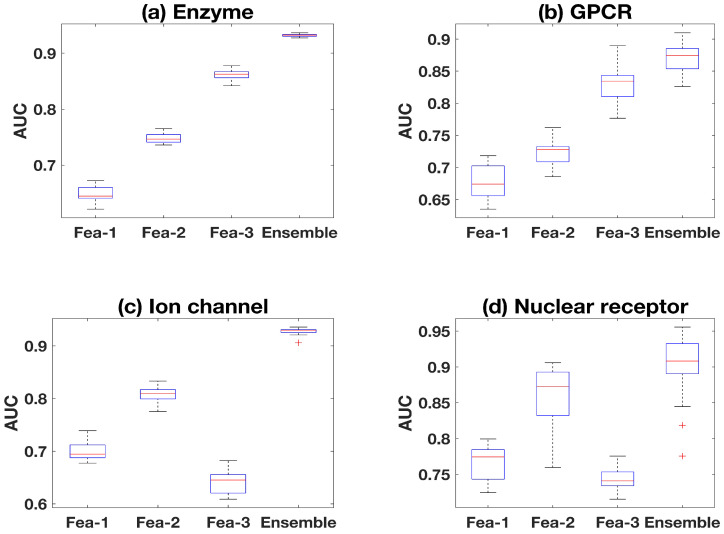
The predictions for new targets. The experiments are based on different targets of training set, validation set and test set.

The ROC curve is shown in Figure 3, which represents the predictions in the validation set and the test set. All DT-pairs containing these drugs are omitted from the training set. Similarly, predictions on different targets are shown in Figure A1. It is shown that the prediction results of the test sets are very close to those of the validation set, which proves that there are no overfitting problems in this work. More evaluation information about these predictions is listed in Table 3.

### 3.2. Comparison with State-of-the-Art Methods

**Figure 4:**
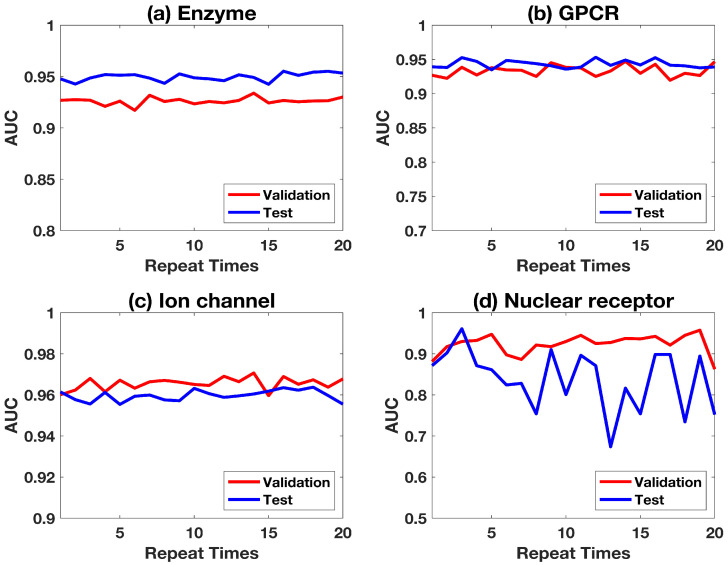
Fluctuations of AUCs in four sub-datasets.

Various methods based on the gold standard dataset are compared. Table 4 shows the average results of the proposed method, and compares four feature vector-based algorithms based on the same dataset, such as that by Wang et al., Multi-scale Features Deep Representations (MFDR), Cao et al., and FRnet-DTI [1,9,25,38]. In these methods, the predictions were obtained by 5-fold cross-validation which were the same as our method. Wang et al. used the stacked autoencoder of deep learning based on the drug molecular structure and protein sequence to predict interactions between drugs and targets. Based on the large-scale drug/target features reconstructed by the autoencoder, SVM is used to predict drug–target interactions in the MFDR method. Cao et al. predicted interactions between the drugs and the target proteins according to the MACCS substructure fingerprint of the drug and the amino acid composition, Composition (C), Transformation (T), and Distribution (D) of the target protein. FRnet-DTI is composed of two convolutional neural networks, FRnet-Encode and FRnet-Predict, for feature manipulation and classification. Except for MFDR, other methods only segment the DT-pairs, and do not consider whether there are drugs that have been trained in the test set; we reproduce these models based on the algorithm in their original paper, and test the drug segmentation test set mentioned in this proposed work. In addition, considering that the negative samples of these algorithms are based on random sampling, we also verify the negative samples of the test set (ran-proposed), and the results are shown in the Table 4. The results show that this method has the best prediction effect in GPCR and ion channel. In enzymes, the predicted results of FRnet-DTI were only 1.3% higher than that of the proposed method. Considering the FRnet-DTI algorithm using two convolutional neural networks for feature extraction and prediction, this method is simple to implement and has closed results. For nuclear receptors, the average results are poor with all the compared algorithms, which may be due to the small dataset and lack of enough training information. The lack of information in nuclear receptor also makes the results unstable, as shown in Figure 4. The predictions based on random sampling (ran-proposed) are also comparable with other methods. In Table 4, the best prediction for each sub-dataset is marked as bold.

## 4. Discussion

### 4.1. Robustness of Prediction

Robustness of the proposed method is discussed. To show the effectiveness and stability of the proposed algorithm, the experiments were carried out 20 times, and the fluctuations of AUCs are shown in Figure 4. It can be seen that, except for the nuclear receptor, the other three sub-datasets have stable predictions in both the validation set and test set.

### 4.2. Weight Optimization of Ensemble Models

**Figure 5:**
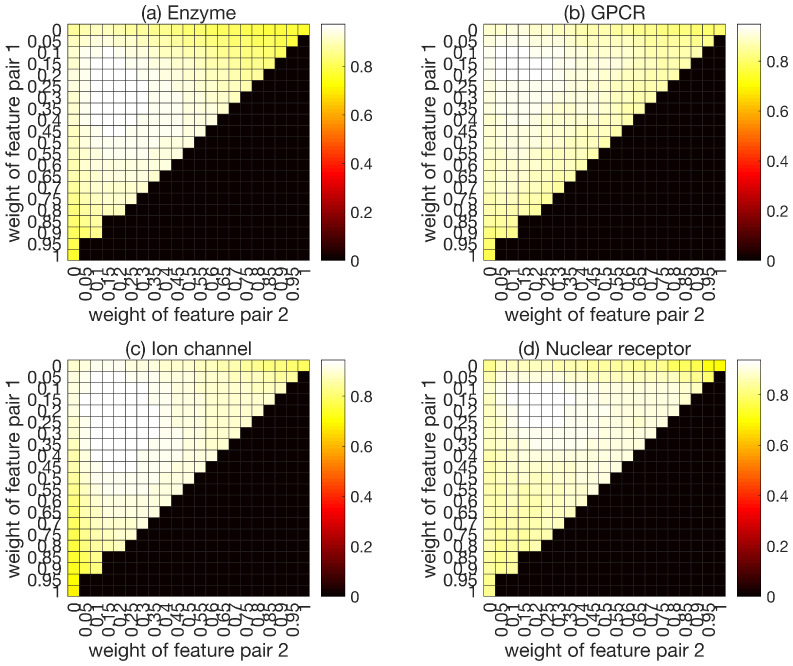
Heatmap of various weights.

The weights of different feature pairs are optimized to obtain better predictions. AUC is the evaluation criteria used in the optimization process. In the process of optimization, $w_{1}\left(0\leq w_{1}\leq 1\right)$, $w_{2}\left(0\leq w_{2}\leq \left(1-w_{1}\right)\right)$ and $w_{3}\left(w_{3}=1-w_{1}-w_{2}\right)$ represent the weights of feature pair 1, feature pair 2, and feature pair 3, respectively. It can be seen from Figure 5 that the prediction results vary with the different weight sequences, which proves the rationality of the Ensemble-MFP algorithm in this work. The maximum predicted results correspond to the optimized weight sequence ($w_{1}$ = 0.1, $w_{2}$ = 0.2, $w_{3}$ = 0.7).

### 4.3. Comparison between Ensemble Models and Individual Model

**Figure 6:**
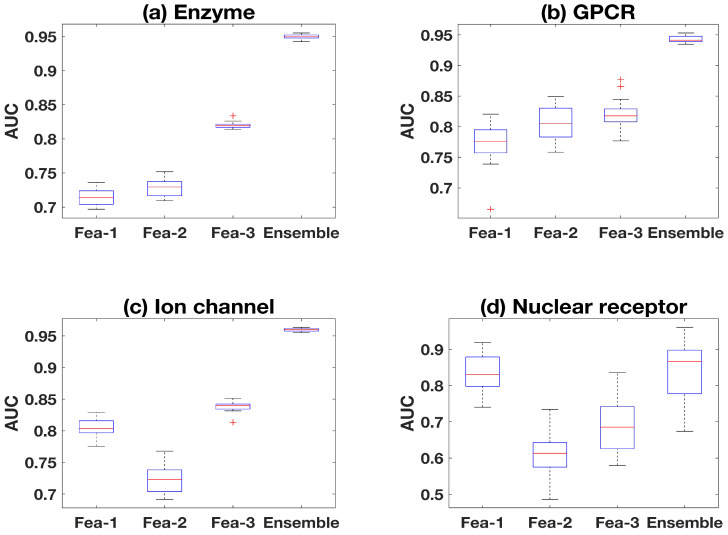
Comparison between Ensemble Models and Individual Model.

**Figure A2:**
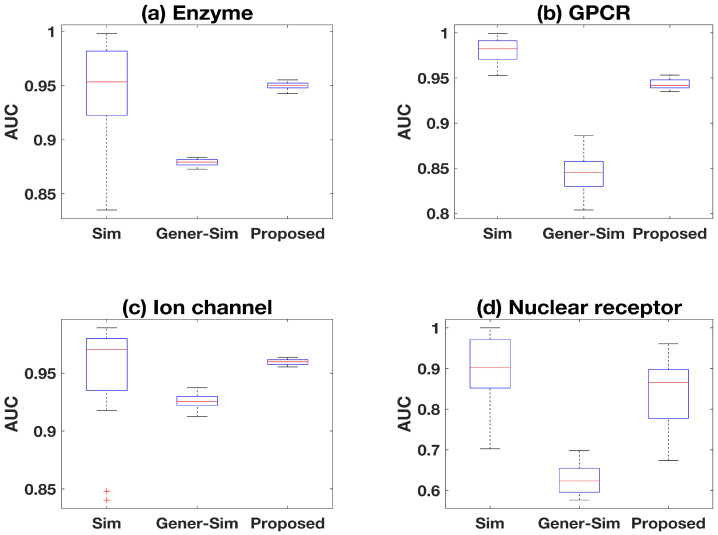
Comparison between simple connection of feature pairs and Ensemble-MFP.

It is shown that the prediction results based on the *Ensemble* models of multiple feature pairs are better than the individual feature pair model in the test set. For each sub-dataset in Figure 6, *Ensemble* represents the predictions based on the Ensemble-MFP method, and *Fea-1*, *Fea-2*, and *Fea-3* represent the results based on only feature pair 1, feature pair 2, and feature pair 3, respectively. In order to make the comparison reliable, all the positive and negative samples used in the training set, validation set, and test set in this part are the same. It is shown that the result of ensemble models is better than that of the individual model with each feature pair, which proves the superiority of the ensemble design. In addition, even if the multiple feature pairs used in the ensemble model are connected to form longer features with weights, better prediction results cannot be obtained, because the ensemble model can simulate more general negative samples (Figure A2).

### 4.4. External Validation

External validation dataset is used to demonstrate the effectiveness of the proposed method. The datasets used in DeepDTI [21], which was extracted based on DrugBank, is used for external validation. At the same time, the independent dataset extracted from the Drug Mechanism of ChEMBL, retaining the inhibitors and Homo sapiens, is tested [39]. After removing the same drugs of the gold standard dataset in the model training, two external datasets were tested with random negative samples. In Table 5, “DeepDTI” denotes the results in their original paper, “proposed-DeepDTI” and “proposed-ChEMBL”, which represent the results on the two external validation datasets based on the proposed method. The results in Table 5 shows the effectiveness of our proposed method, and TPR and TNR represent the True Positive Rate (*TP*/(*TP* + *FN*)) and True Negative Rate (*TN*/(*TN* + *FP*)), respectively. In addition, two predicted drug–target pairs were demonstrated as interactions (Lysine (DB00194) interacts with SLC7A4 (O43246) [40,41], and Micafungin (DB01141) interacts with FKSA (A2QLK4)) [42,43].

## 5. Conclusions

In this work, an algorithm based on the Ensemble models of Multiple Feature Pairs (Ensemble-MFP) is proposed for drug–target interaction predictions. Three models are obtained through three feature pairs, and the weights of the models are optimized on the validation set and applied on the test set. In order to make the model more general, the negative samples in the validation set/test set are collected randomly from three negative sets, which are extracted based on the Euclidean distance of three feature pairs. It is shown that, compared with the individual model of the single feature pair on the test set in the algorithm, the prediction effects of the Ensemble-MFP are better, which proves the effectiveness of the method. In addition, according to the external validation and demonstration results of the predicted DT pairs, the proposed method has a significance contribution on the drug design.

The algorithm can be further extended based on the details of more feature pairs. For the sake of simplicity, only three feature pairs are studies in this work. In addition, more feature pairs can be added to the algorithm. At the same time, according to the drug–target pairs predicted, we believe that our algorithm will supply more potential DT-pairs for wet-lab people, and motivate more researchers to study DTI in depth. Finally, the binary classification method restricts the further development of DTI to a certain extent, which will be the development direction in the future.

## Figures and Tables

**Table 1:** Statistics of gold-standard datasets.

	Enzyme	GPCR	Ion Channel	Nuclear Receptor
Drugs	445	223	210	54
Targets	664	95	204	26
DTIs	2926	635	1476	90
unlabeled DT-pairs	292,554	20,550	41,364	1314

**Table 2:** Feature Pairs used in Ensemble-MFP.

	Drug Descriptor	Dimension	Target Descriptor	Dimension
Feature Pair 1	Estate-FP	79	AAC	20
Feature Pair 2	MACCS-FP	166	APAAC	80
Feature Pair 3	Sub-FP Count	307	QSO	160

**Table 3:** Prediction results of the proposed method.

	Enzyme	GPCR	Ion Channel	Nuclear Receptor
Accuracy (%)	89.92 ± 0.93 ^#^	96.50 ± 0.70	85.01 ± 1.68	84.32 ± 12.44
Precision (%)	90.37 ± 0.93	98.89 ± 0.16	84.90 ± 1.68	91.29 ± 13.70
Recall (%)	100 ± 0.00	97.14 ± 0.96	100.00 ± 0.00	89.68 ± 17.01
F1-scores (%)	94.94 ± 0.72	98.01 ± 0.23	91.83 ± 0.98	90.48 ± 8.72
AUC (%)	95.92 ± 0.39	94.32 ± 0.57	95.97 ± 0.26	83.87 ± 7.38

^#^ The value in the Table means the average ± standard deviation.

**Table 4:** Comparison with state-of-the-art methods on the gold standard dataset. Proposed and ran-proposed represent the predictions with proposed work and random sampling, respectively.

AUC	Enzyme	GPCR	Ion Channel	Nuclear Receptor
Wang et al.	0.916	0.897	0.907	0.775
MFDR	0.969	0.904	0.933	0.886
Cao et al.	0.938	0.839	0.875	0.809
FRnet-DTI	0.972	0.912	0.943	0.872
Proposed	0.959	0.943	0.960	0.839
ran-proposed	0.933	0.908	0.925	0.821

**Table 5:** Predictions on external validation datasets.

	TPR (%)	TNR (%)	Accuracy (%)	AUC (%)
DeepDTI	82.27	89.53	85.88	91.58
proposed-DeepDTI	86.23	88.65	91.69	93.01
proposed-ChEMBL	90.09	93.18	90.57	92.78

## Data Availability

The benchmark dataset used in the method was first introduced by Yamanishi et al., and was collected based on KEGG BRITE, DrugBank, BRENDA and SuperTarget. It can be downloaded freely at http://web.kuicr.kyoto-u.ac.jp/supp/yoshi/drugtarget/, accessed on 1 July 2008, and “Adjacency matrix of the gold standard drug–target interaction data” are used in the proposed method. The gold standard data set had been used in many studies related to drug–target interactions, and it is easy to compare the predicted results. PaDEL-Descriptor is a free software to extract drug descriptors, which is produced by the Pharmaceutical Data Exploration Laboratory and can be downloaded from https://www.winsite.com/, accessed on 12 October 2010. The method adopts version 2.21. PROFEAT is a webservice for computing the characteristics of related proteins on http://bidd.group/, accessed on 12 April 2011. Libsvm version 3.23 is used in this method. It was produced by Lin et al and can be downloaded in https://www.csie.ntu.edu.tw/~cjlin/libsvm/, accessed on 5 May 2003. The index parameter adjustment method with 2 at the bottom is based on the 2016 libsvm practical guide. The algorithm is implemented on MacOS High Sierra (10.13.6) platform by MATLAB R2018b. And the code is available on GitHub (https://github.com/Wangcheng0788/Ensemble-MFP, accessed on 16 June 2021).

## Abbreviations

The following abbreviations are used in this manuscript:

DTI	drug–target Interaction
Ensemble-MFP	Fusion of Multiple Feature Pairs
AUC	Area Under the Curve for ROC
ROC	Receiver Operating Characteristics
PU	Positive-Unlabeled problems
GPCR	G Protein-Coupled Receptors
SVM	Support Vector Machines
RBF	Radial Basis Function

## Appendix A. Predictions for New Targets

Similar prediction results are obtained when the target proteins do not appear repeatedly in training set, validation set and test set, which proves the effectiveness on new target protein prediction of the Ensemble-MFP. The prediction results are shown in Figure A1, and *Ensemble* represents the results of proposed method, *Fea-1*, *Fea-2*, *Fea-3* denote the predictions based on the model of feature pair 1, feature pair 2 and feature pair 3 respectively.

## Appendix B. Comparison between Simple Connection of Feature Pairs and Ensemble-MFP

The prediction effect of three feature pairs on simple combination is worse than that of different models trained by three features. The difference between the two methods lies in the construction of negative samples. The simple connection of multiple feature pairs is similar to the result of a single feature pair, and cannot predict more general negative samples. In contrast, the method proposed in this work, on the one hand, simulates more general negative samples to a certain extent by combining and randomly selecting three groups of negative samples; on the other hand, by optimizing the weights, the model can get better prediction in general samples. We test and demonstrate several different experimental situations, including: (1) the case of simple feature connection, we test the negative samples based on Euclidean distance screening (Sim); (2) the case of simple feature connection, the more general negative samples designed in this work are tested (Gener-Sim); (3) the proposed results (Proposed). For the sake of fairness, in case (2), we also optimize the weights of each simply connected features. It can be seen from the Figure A2 that although the result of negative samples based on single feature extraction is very good (Sim), it is difficult to achieve good prediction for more general negative samples (Gener-Sim) with longer feature forms. In contrast, the weighted method mentioned in this work is better.

## Appendix C. Predicted Drug–Target Pairs

The top ten predicted drug–target pairs in GPCR are listed in Table A1. We use the proposed algorithm to predict the drug–target pairs in the test set and rank them according to the decision values. After several times of algorithm prediction, 10 groups of drug–target pairs with high decision scores were selected, and the information was input into the drug database (DrugBank) for query. Through the query, we found that the two groups of predicted drug–target pairs recorded as unlabeled drug–target pairs in 2008 had interaction records in the database. In addition, Trimipramine (D00394) interacts with HTR1A and HTR1B, so there may be interaction between D00394 and HTR1F (hsa3355) [44,45,46,47]. In the Table A1, 8 out of 10 pairs of predicted drug targets can be further demonstrated by wet-lab people. The results show that this method can effectively predict new drugs and is of great significance for drug development.

**Table A1:** Top ten drug–target pairs predicted in GPCR.

GPCR	Drug	Drug Name	Target	Gene Name	Record of Database	Score
1	D00394	Trimipramine	hsa3355	HTR1F	-	1.0973
2	D00563	Mirtazapine	hsa3355	HTR1F	-	1.0623
3	D00394	Trimipramine	hsa154	ADRB2	-	1.0365
4	D02566	Maprotiline	hsa3355	HTR1F	-	1.0350
5	D00563	Mirtazapine	hsa154	ADRB2	-	0.9933
6	D00483	Propranolol	hsa3355	HTR1F	-	0.9854
7	D00394	Trimipramine	hsa147	ADRA1B	DrugBank	0.9768
8	D00394	Trimipramine	hsa1128	CHRM1	-	0.9765
9	D00563	Mirtazapine	hsa147	ADRA1B	-	0.9696
10	D00394	Trimipramine	hsa3350	HTR1A	DrugBank	0.9666
